# Supplementary material for: A Small Step Toward Generalizability: Training a Machine Learning Scoring Function for Structure-Based Virtual Screening
Source: J Chem Inf Model. 2023 May 11;63(10):2960–74. doi: 10.1021/acs.jcim.3c00322 (PMC10207375; doi:10.1021/acs.jcim.3c00322)
Supplement: Supplementary file 1 — ci3c00322_si_001.pdf [file ci3c00322_si_001.pdf]

Supporting Information

A Small Step Toward Generalizability: Training a Machine Learning Scoring Function for Structure-Based Virtual Screening

Jack Scantlebury[1,*], **Lucy Vost**[1,*], **Anna Carbery**[1,2], **Thomas E. Hadfield**[1], **Oliver M. Turnbull**[1], **Nathan Brown**[3], **Vijil Chenthamarakshan**[4], **Payel Das**[4], **Harold Grosjean**[5], **Frank von Delft**[2,6,7,8], and **Charlotte M. Deane**[1,**]

[1] *Department of Statistics, University of Oxford, Oxford, UK*

[2] *Diamond Light Source Ltd., Harwell Science and Innovation Campus, Didcot, UK*

[3] *Healx, Charter House, Cambridge, UK; at time of work: BenevolentAI, London, UK*

[4] *IBM Thomas J. Watson Research Center, New York, USA*

[5] *Structural Genomics Consortium, University of Oxford, Oxford, UK*

[6] *Centre for Medicines Discovery, University of Oxford, Oxford, UK*

[7] *Department of Biochemistry, University of Johannesburg, Johannesburg, South Africa*

[8] *Research Complex at Harwell, Harwell Science and Innovation Campus, Didcot, UK*

[*] *Authors contributed equally*

[**] *deane@stats.ox.ac.uk*

1. Equivariance and Invariance

A function $\psi : X \to Y$ is *invariant* with respect to a group of transformations $G : X \to X$ if:

$$\psi(g(x)) = \psi(x) \quad \forall g \in G \quad \forall x \in X \tag{1}$$

ψ is *equivariant* with respect to G if there is a transformation $T : Y \to Y$ such that:

$$\psi(g(x)) = T(\psi(x)) \quad \forall g \in G \quad \forall x \in X \tag{2}$$

In other words, if ψ is equivariant under G, applying elements of G to x will apply an analogous transformation to the output in Y. The order in which the transformations are applied does not matter to the end result.

While CNNs offer invariance to elements of the group $G = T(n)$, the set of all translations in n-dimensional space, they lack invariance to the group of all roto-translations, $SE(n)$. This means that rotating the protein-ligand input in 3D space may produce different predictions, behavior which is undesirable. Usually, the way to combat this is to augment training data by assigning random rotations to the inputs, but this does not achieve true rotational invariance. For a more thorough introduction to symmetry groups and equivariance, see references.[1–4]

Group-equivariant neural networks are a new class of deep learning model which offer invariance to various symmetry groups. They do this by using a series of equivariant layers followed by one or more invariant layer. For CNNs, these include Group Equivariant CNNs[1] and steerable CNNs.[5] CNNs still have the fundamental problem of a discrete input space and an extremely large input space and parameter count (a $48 \times 48 \times 48$ grid with 22 channels is more than 2.4×10^6 floating point numbers). If we view molecules as graphs, however, we can apply group-equivariant GNNs to the problem of pose predictions.

For these reasons, we use an architecture based on *E(n)*-Equivariant Graph Neural Network (EGNN) layers[2] for pose selection. The $E(n)$ symmetry group contains all elements from $SE(n)$, as well as the group of all reflections; EGNN layers are also permutation-equivariant, meaning that the network is invariant to the order of the input vertices.

2. PointVS: Methods

2.1 Model

A graph is a natural way of representing a molecule, and attribution to individual inputs—namely atoms—is an intuitive process when using graph neural networks (GNNs). We use an architecture based on *E(n)*-Equivariant Graph Neural Network (EGNN) layers.[2] The $E(n)$ symmetry group contains all elements from $SE(n)$, as well as the group of all reflections; EGNN layers are also permutation-equivariant, meaning that the network is invariant to the order of the input vertices.

PointVS is a lightweight $E(n)$-equivariant graph neural network layer model, consisting of an initial projection to take the number of node features from 12 to 32, then 48 EGNN layers, followed by a global average pooling of the final node embeddings. This is followed by a sigmoid layer which gives a label $y \in [0,1]$ during the first stage of training on pose prediction. During finetuning on affinity data, this final layer is replaced by a randomly initiated fully connected layer and ReLU activation, which outputs $\mathbf{y} \in (\mathbb{R}^+)^3$. This architecture includes residual connections for node features to allow for easier gradient flow and a richer combination of early and late representations. It also uses a shallow neural network as an attention mechanism,[6] which learns to score network edges—in this case, representing atomic interactions—by their importance.

2.2 Training

All models were trained for 10 epochs with a batch size of 32, using the Adam optimizer and cosine warm restarts with a maximum learning rate of 8×10^{-4} and a weight decay of 10^{-4}. For classification models, the selected model is the one with the best Top-1 value on the test set; for regression models, the selected model is the one with the highest Pearson's Correlation Coefficient (PCC) for affinity on the PDBBind Core Set.

For regression models with pretraining, once the classification task is complete, the best model is selected and the final linear layer with sigmoid is replaced by a multi-target regression, where the target is either pK_d, pK_i or pIC_{50}. The labels for the two types of affinity data not present in the training set are ignored for calculating loss, so those weights are unaffected.

2.3 Datasets

The Redocked Set

Training for pose classification uses the same training data as the Redocked model outlined by McNutt *et al.*[7] In previous work by the same authors,[8] ligands from the Pocketome v17.12[9] were redocked using AutoDock Vina into their cognate receptors, with an RMSD calculated between the redocked poses and the crystal pose. Sub-2 Å poses were labeled as active poses, with the rest labeled as inactive. Further negative examples were generated adversarially: a gnina CNN trained on the original redocked data set was used to optimize docked poses. Optimized poses that were scored highly (> 0.9) by the trained CNN but were more than 2 Å RMSD from the crystal pose were added as inactive poses. In total, there are 52,385 active and 734,417 inactive poses in the version of Redocked2020 used here, which we have shortened to Redocked.

PDBBind Refined: gninaSet$_{Pose}$ Set

The PDBBind refined set v.2019[10] is a carefully selected set of high-quality protein-ligand complexes, complete with binding affinities. In their gnina 1.0 paper,[8] the authors took the 4,852 unique protein-ligand complexes, removing solvents and any other molecules besides the protein and the ligand, as well as removing structures with ligands that do not have a mass between 150 and 1,000 Da. This filtered, preprocessed version of the refined set is hosted by the authors of gnina 1.0[8] at `https://bits.csb.pitt.edu/files/gnina1.0_paper`. Gnina was used with default settings, excepting `--num_modes=50`, `--min_rmsd_filter=0.0`, and the use of the standard Vina scoring function in refinement and ranking rather than a CNN.

Of the 4,260 filtered structures, the 441 which are not used in training the 'redocked' gnina model are selected as the test set for consistency with that work, which we call gninaSet$_{Pose}$. Up to 50 poses are reranked by our GNN (some of which may be very similar), after which the standard 1.0 Å RMSD filter is applied to get a final pose ranking.

PDBBind General and Core Sets

The PDBBind v.2020 General set contains protein-ligand complexes and binding affinities curated from the PDB.[11] The affinities are in the form of pK_d, pK_i or pIC_{50}, and this was the training set for affinity regression. The PDBBind Core set, also known as the CASF-2016 test set for affinity regression, comprises 285 high-quality crystal structures, as well as their binding affinities,[12] and is a subset of the General set. This is the test set for regression (*scoring power*).

3. Assessing Attribution Methods

As described in the paper, we use three methods for attribution: atom masking, bond masking, and edge attention

analysis. We showed that when carrying out atom masking with gnina,[8] bond masking with InteractionGraphNet (IGN)[13] and edge attention analysis with PointVS, the method that extracted important protein atoms in most agreement with PLIP, the protein-ligand interaction profiler.[14] To learn to what extent this could be accredited to the method used for attribution, we also carried out atom masking and bond masking on PointVS, the results of which are given in Table 1.

Attribution	MLBSF	ρ_5	ρ_{10}
Edge attention	PointVS	0.640	0.788
Atom masking	gnina	0.234	0.093
	PointVS	0.152	0.169
Bond masking	IGN	0.209	-0.071
	PointVS	0.140	0.122

Table 1. Mean rank correlation calculated between the scores of the top five (ρ_5) and top ten (ρ_{10}) ranked protein atoms by PointVS, gnina, and InteractionGraphNet (IGN) and the distance between them and the nearest polar ligand atom. The means were calculated over a subset of 20 randomly selected bound structures from the PDBBind Core set (see SI for PDB IDs).

4. Hotspots API: Calculation and Processing of Hotspots

For a given protein target, the API calculates acceptor, donor, and apolar atomic propensity maps using SuperStar.[15] This tool defines a grid covering the protein with points spaced 0.5Å apart, and then using data from the Cambridge Structural Database (CSD),[16] assigns a propensity for the three atomic probe types—acceptor, donor, and apolar—to each point. These scores are assigned based on the principle that if a positive interaction between two groups at a certain distance and angle is made, then it will occur more frequently in structures stored in the CSD, and thus be assigned a higher propensity score. These grid point scores are then weighted according to how buried in the protein they are so that more buried sites are favored.

Further scores are then calculated with small chemical probes consisting of aromatic rings with an acceptor, donor, or apolar atom in the substituent position. The three weighted propensity grids are sampled by their corresponding probe: a given probe is translated to all grid points with scores above a threshold of 15, and randomly rotated 3000 times around the center of the substituent atom. For each pose, the probe atom scores are read from their corresponding grids, and the probe score is found as the geometric mean of the scores of the probe's atoms. Once complete, the sampled probe scores are assigned to an output grid.

To process the donor and acceptor grids, we follow the protocol outlined in Hadfield *et al.*[17] A greedy clustering algorithm is employed. A cluster is initialized as a random single point, and all points less than 1Å away that are not part of another cluster are added. Then, for each point added, this process is repeated, so all remaining unclustered points within 1Å are added. This process continues until there are no unclustered points within 1Å of the points forming the cluster. Once a cluster is finalized, a new cluster is defined by selecting another single unclustered point. This process is repeated until all points have been assigned to a cluster. The centroids of these clusters are defined as the mean position of the points constituting them. Clusters comprising less than eight points are removed, as well as those with centroids outside of an apolar region (this is assessed using the apolar grid). The remaining clusters are then allocated a score consisting of the number of points comprising them, and output alongside their associated scores as the acceptor and donor hotspot maps for a given protein target.

5. Structure IDs

The bound fragment structures used for all three targets (Mpro, Mac1 and NSP14) are listed in Table 2. These datasets are all available to download from the fragalysis platform.[18–21]

The PDB IDs of the randomly selected subset of 20 structures from the PDBBind core set used in section 3.4.2 are 3URI, 4DE1, 4DLI, 3N76, 4M0Z, 3TWP, 1VSO, 3NQ9, 3UEU, 3AO4, 3JVR, 2BRB, 1H23, 1E66, 2FVD, 2ZB1, 4K18, 3OE5, 3GE7, 3RR4, 3UI7, 4JSZ, 3QQS, 2VKM, 4MGD, 2XII, 2QE4, 1Y6R.

6. Mpro: Hotspot Map Dependency on Fragment Screen Size

We randomly generate sets of bound structures of Mpro of various sizes, and perform attribution on them to obtain PointVS hotspots. The results from this are shown in Fig 4.

As another assessment of the consistency of PointVS hotspots, we assert that the top five hotspots found when PointVS is given all of the Mpro fragment screen structures in Table 1 can be considered the 'ground truth' top five hotspots. We then carry out attribution on various sizes of randomly sampled sets of bound structures, as above, and record how many of the top five hotspots for a given

Mpro			Mac1		NSP14
x10474_0A	x10484_0A	x10494_0A	DLS-EU0569_0A	DLS-X0626_0A	x1203_0D
x10898_0A	P0906_0B	x11543_0A	DLS-X0724_0A	DLS-X0689_0A	x1088_0D
x2964_0A	x11426_0A	x2646_0A	DLS-X0591_0A	DLS-X0711_0A	x1201_0D
x11372_0A	x10604_0A	x10559_0A	DLS-X0681_0A	DLS-EU0034_0A	x1316_0D
x11233_0A	x12682_0A	x11764_0A	DLS-X0895_1A	DLS-EU0087_0A	x1448_0D
x10473_0A	x10889_0A	x2971_0A	DLS-X0910_0A		x1412_0D
x10327_0A	P1470_0A	x11159_0A	DLS-X0587_0A		x1181_0D
x12740_0A	x10723_0A	x2908_0A	DLS-X0421_0A		x1199_0D
x10888_0A	x10248_0A	x11532_0A	DLS-EU0342_0A		x1197_0D
x10371_0A	P0045_0B	x0678_0A	DLS-X0844_0A		x1384_1D
x10606_0A	x10756_0A	x11164_0A	DLS-EU0815_0A		x1486_0D
x11743_0A	x10995_0A	P0045_0A	DLS-EU0291_0A		x1245_0D
x2912_0A	x10555_0A	x3108_0A	DLS-X0299_0A		x1541_0D
x10565_0A	x2572_0A	x11339_0A	DLS-X0471_0A		x1603_0D
x10422_0A	P0906_0A	x11564_0A	DLS-X0895_0A		x1236_2D
x10421_0A	P2007_0B	x10789_0A	DLS-EU0238_0A		x1282_0D
P1073_0A	x3359_0A	P2001_0B	DLS-X0766_0A		x1641_0D
x10488_0A	x10387_0A	x2608_0A	DLS-X0926_0A		x1078_0D
x11427_0A	x0107_0A	x10856_0A	DLS-EU0056_0A		x1212_1D
P0145_0B	x10201_0A	x10314_0A	DLS-X0592_0A		
x10942_0A	x11271_0A	x11513_0A	DLS-X0967_0A		
x10022_0A	x2581_0A	x11540_0A	DLS-EU0144_0A		
x11473_0A	x10377_0A	x2600_0A	DLS-X0722_0A		
x0434_0A	x10395_0A	x10598_0A	DLS-EU0704_0A		
P1470_0B	x10329_0A	x11041_0A	DLS-EU0844_0A		
x12719_0A	x10178_0A	x11417_0A	DLS-EU0481_0A		
x10396_0A	x12300_0A	x10638_0A	DLS-X0969_0A		
z7vh8_0A	x10324_0A	x10334_0A	DLS-EU0241_0A		
x11368_0A	P0884_0A	x10392_0A	DLS-X0962_0B		
P2007_0A	x11346_0A	x11708_0A	DLS-X0742_0A		
x11231_0A	x2649_0A	x11318_0A	DLS-EU0172_0A		
x10906_0A	x11641_0A	x10019_0A	DLS-X0918_0A		
x3080_0A	x10800_0A	x11428_0A	DLS-X1018_0A		
x11560_0A	x10996_0A	x10733_0A	DLS-X0962_0A		
x11013_0A	x10417_0A	x11475_0A	DLS-X0900_0A		
x10566_0A	x10247_0A	x10478_0A	DLS-X0685_0A		
P2001_0A	x3298_0A	x11485_0A	DLS-X0962_1A		
x11809_0A	x3366_0A	x10575_0A	DLS-X0805_0A		
x10679_0A	x2643_0A	P0145_0A	DLS-X0142_0A		
x10801_0A	x11488_0A	x10787_0A	DLS-X0104_0A		
P0925_0A	x11317_0A	x2569_0A	DLS-X0852_0B		
x11225_0A	x10889_1A	x11044_0A	DLS-X0918_1A		
x10338_0A	x10476_0A	x11723_0A	DLS-EU0716_0A		
x10359_0A	x11562_0A	x10322_0A	DLS-EU0123_0A		
x2193_0A	x10900_0A	x11354_0A	DLS-X0905_0A		
x10423_0A	x2562_0A	x12321_0A	DLS-X0910_1A		
x11557_0A	x11801_0A	x11025_0A	DLS-X0933_0A		
x10645_0A	x10237_0A	x10834_0A	DLS-X0837_0A		
x10626_0A	x11011_0A	x11507_0A	DLS-EU0293_0A		
x10236_0A	x10535_0A	x10976_0A	DLS-EU0424_0A		
x11186_0A	x10610_0A		DLS-EU0046_0A		

Table 2. Fragalysis identification codes of the structures supplied to PointVS to obtain hotspots.

random sample coincide with the ground truth top five hotspots. The results from this are shown in Fig 1. We see that increasing the size of the randomly selected set of bound structures results in increased agreement with the ground truth hotspots. However, we also note that this effect drops off when the size of the sets of bound structures reaches 30, implying that after this point, increasing the size of a fragment screen would have little benefit on the quality of the top five hotspots produced by PointVS. This is a promising sign of the applicability of the PointVS hotspots, as Mpro is very unusual in having such a large number of fragment structures available.

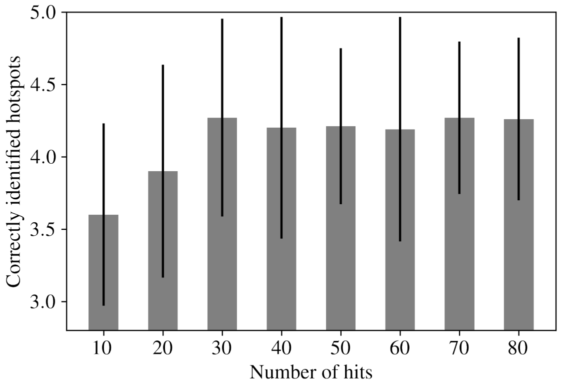

Fig. 1. The average number of hotspots found by PointVS using different numbers of fragment structures of Mpro that agree with those found using the full set of 152 fragment structures, with error bars showing the standard deviation of the number of ground truth hotspots identified.

7. Fragment Elaboration: Numbers of Elaborations Generated

Hotspots for the targets Mac1 and NSP14 are tested on 35 and 30 fragments respectively. The number of fragments successfully elaborated for each hotspot and the mean number of elaborations for each fragment are shown in Tables 3a and b.

8. FGFR1 Case Study

To demonstrate the potential of the PointVS hotspot maps for real-world fragment-to-lead campaigns, we present a case study on a well-studied set of drug targets: the fibroglast growth factor receptors. Aberrant activation of these receptors is associated with a wide variety of cancers, making them a promising druggable target. In 2019, a collaboration between Astex and Newcastle University resulted in erdafitnib:[22] a drug that has since been FDA approved, making it the third fragment-derived drug to reach this stage.

In the process of designing erdafitnib, the collaborators heavily relied on a fragment screen performed on the target in 2008. A fragment found in this screen, shown in Fig 2, was found to have high potency, and was used as a starting point in the design process.

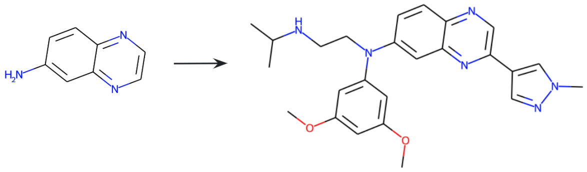

Fig. 2. Left, the fragment used as a starting point in the development of erdafitnib, shown on the right.

In Patani *et al.*,[23] two hydrogen bonds are found to form between erdafitnib and the protein. We investigated whether we could identify the protein atoms forming these bonds as important using either PointVS or the API hotspots.

The API placed one donor hotspot 1.5Å away from the location of one of the erdafitnib atoms that forms a hydrogen bond with a protein atom. However, it did not recognize the point the second binding ligand atom is found at as important.

To obtain the PointVS hotspots, we performed attribution on 18 of the bound structures of FHFR1 available in the PDB.[11] These bound structures contain compound-like molecules rather than fragments, so we filtered them to ensure that none had a Tanimoto similarity of greater than 0.7 to erdafitnib. This resulted in us selecting the structures corresponding to the following PDB codes: 4NKS, 4NK9, 1AGW, 3C4F, 4NKA, 4V01, 4V05, 5B7V, 5A46, 4UWB, 3RHX, 1FGI, 5A4C, 4UWC, 5VND, 6P69.

We found that PointVS correctly identified both of the protein atoms that form hydrogen bonds with ligand atoms as important, ranking them as the third and sixth most important atoms for binding.

To provide an example of how knowledge of these two hotspots could be used, we supplied one of them to STRIFE to demonstrate that molecules similar to erdafitnib could be generated. An example of an elaboration that recovered the same binding mode as erdafitnib is shown in Fig 3.

	PointVS		Hotspots API	
Rank	Fragments successfully elaborated	Elaborations per fragment	Fragments successfully elaborated	Elaborations per fragment
1	21	217	15	189
2	12	187	10	183
3	13	223	11	207
4	11	192	7	158
5	5	207	12	179
6	9	176	15	162
7	6	239	21	220
8	2	222	11	197
9	0	0	0	0
10	1	218	8	210

a

	PointVS		Hotspots API	
Rank	Fragments successfully elaborated	Elaborations per fragment	Fragments successfully elaborated	Elaborations per fragment
1	14	194	2	237
2	3	196	23	218
3	24	215	24	195
4	23	186	14	154
5	14	183	0	0
6	23	186	24	195
7	17	194	0	0
8	5	196	4	116
9	1	89	27	199
10	6	205	13	181

b

Table 3. The number of fragments that were successfully elaborated on using PointVS and API hotspots for Mac1 (a) and NSP14 (b), and the mean number of elaborations generated per fragment.

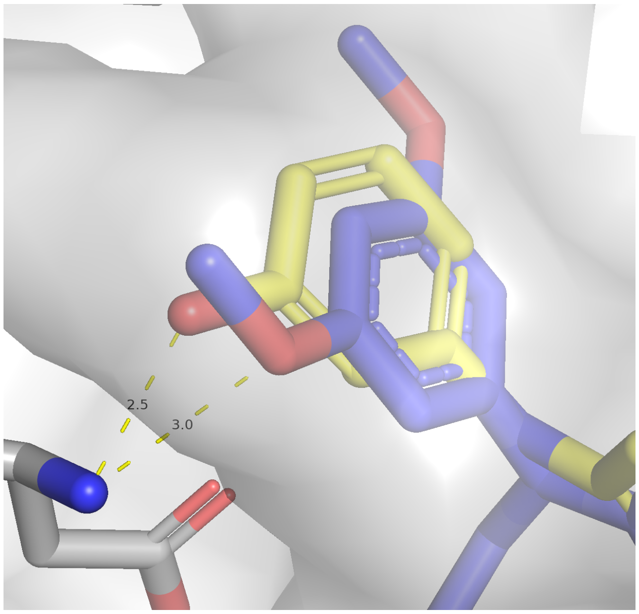

Fig. 3. Elaboration generated by STRIFE using the PointVS hotspots (yellow), that recovers the same hydrogen bond as erdafitnib, shown in blue (PDB ID: 5EW8[23]).

BIBLIOGRAPHY

[1] T. S. Cohen and M. Welling, "Group equivariant convolutional networks," *CoRR*, vol. abs/1602.07576, 2016.

[2] V. G. Satorras, E. Hoogeboom, and M. Welling, "E(n) equivariant graph neural networks," 2021.

[3] M. Hutchinson, C. L. Lan, S. Zaidi, E. Dupont, Y. W. Teh, and H. Kim, "Lietransformer: Equivariant self-attention for lie groups," *CoRR*, vol. abs/2012.10885, 2020.

[4] M. Finzi, S. Stanton, P. Izmailov, and A. G. Wilson, "Generalizing convolutional neural networks for equivariance to lie groups on arbitrary continuous data," 2020.

[5] T. S. Cohen and M. Welling, "Steerable cnns," *CoRR*, vol. abs/1612.08498, 2016.

[6] A. Vaswani, N. Shazeer, N. Parmar, J. Uszkoreit, L. Jones, A. N. Gomez, L. u. Kaiser, and I. Polosukhin, "Attention is all you need," in *Advances in Neural Information Processing Systems* (I. Guyon, U. V. Luxburg, S. Bengio, H. Wallach, R. Fergus, S. Vishwanathan, and R. Garnett, eds.), vol. 30, Curran Associates, Inc., 2017.

[7] P. G. Francoeur, T. Masuda, J. Sunseri, A. Jia, R. B. Iovanisci, I. Snyder, and D. R. Koes, "Three-dimensional convolutional neural networks and a cross-docked data set for structure-based drug design," *Journal of Chemical Information and Modeling*, vol. 60, no. 9, pp. 4200–4215, 2020. PMID: 32865404.

[8] A. T. McNutt, P. Francoeur, R. Aggarwal, T. Masuda, R. Meli, M. Ragoza, J. Sunseri, and D. R. Koes, "Gnina 1.0: molecular docking with deep learning," *Journal of Cheminformatics*, vol. 13, no. 1, 2021.

[9] I. Kufareva, A. V. Ilatovskiy, and R. Abagyan, "Pocketome: an encyclopedia of small-molecule binding sites in 4d," *Nucleic Acids Research*, vol. 40, no. D1, pp. D535–D540, 2011.

[10] Z. Liu, M. Su, L. Han, J. Liu, Q. Yang, Y. Li, and R. Wang, "Forging the basis for developing protein–ligand interaction scoring functions," *Accounts of Chemical Research*, vol. 50, no. 2, pp. 302–309, 2017. PMID: 28182403.

[11] S. K. Burley, C. Bhikadiya, C. Bi, S. Bittrich, L. Chen, G. V. Crichlow, C. H. Christie, K. Dalenberg, L. Di Costanzo, J. M. Duarte, S. Dutta, Z. Feng, S. Ganesan, D. S. Goodsell, S. Ghosh, R. K. Green, V. Guranović, D. Guzenko, B. P. Hudson, C. L. Lawson, Y. Liang, R. Lowe, H. Namkoong, E. Peisach, I. Persikova, C. Randle, A. Rose, Y. Rose, A. Sali, J. Segura, M. Sekharan, C. Shao, Y.-P. Tao, M. Voigt, J. D. Westbrook, J. Y. Young, C. Zardecki, and M. Zhuravleva, "RCSB Protein Data Bank: powerful new tools for exploring 3D structures of biological macromolecules for basic and applied research and education in fundamental biology, biomedicine, biotechnology, bioengineering and energy sciences," *Nucleic Acids Research*, vol. 49, pp. D437–D451, 11 2020.

[12] M. Su, Q. Yang, Y. Du, G. Feng, Z. Liu, Y. Li, and R. Wang, "Comparative assessment of scoring functions: The casf-2016 update," *Journal of Chemical Information and Modeling*, vol. 59, no. 2, pp. 895–913, 2019.

[13] D. Jiang, C.-Y. Hsieh, Z. Wu, Y. Kang, J. Wang, E. Wang, B. Liao, C. Shen, L. Xu, J. Wu, D. Cao, and T. Hou, "Interactiongraphnet: A novel and efficient deep graph representation learning framework for accurate protein–ligand interaction predictions," *Journal of Medicinal Chemistry*, vol. 64, p. 18209–18232, Dec 2021.

[14] S. Salentin, S. Schreiber, V. J. Haupt, M. F. Adasme, and M. Schroeder, "PLIP: fully automated protein–ligand interaction profiler," *Nucleic Acids Research*, vol. 43, pp. W443–W447, 04 2015.

[15] M. L. Verdonk, J. C. Cole, and R. Taylor, "Superstar: A knowledge-based approach for identifying inter-

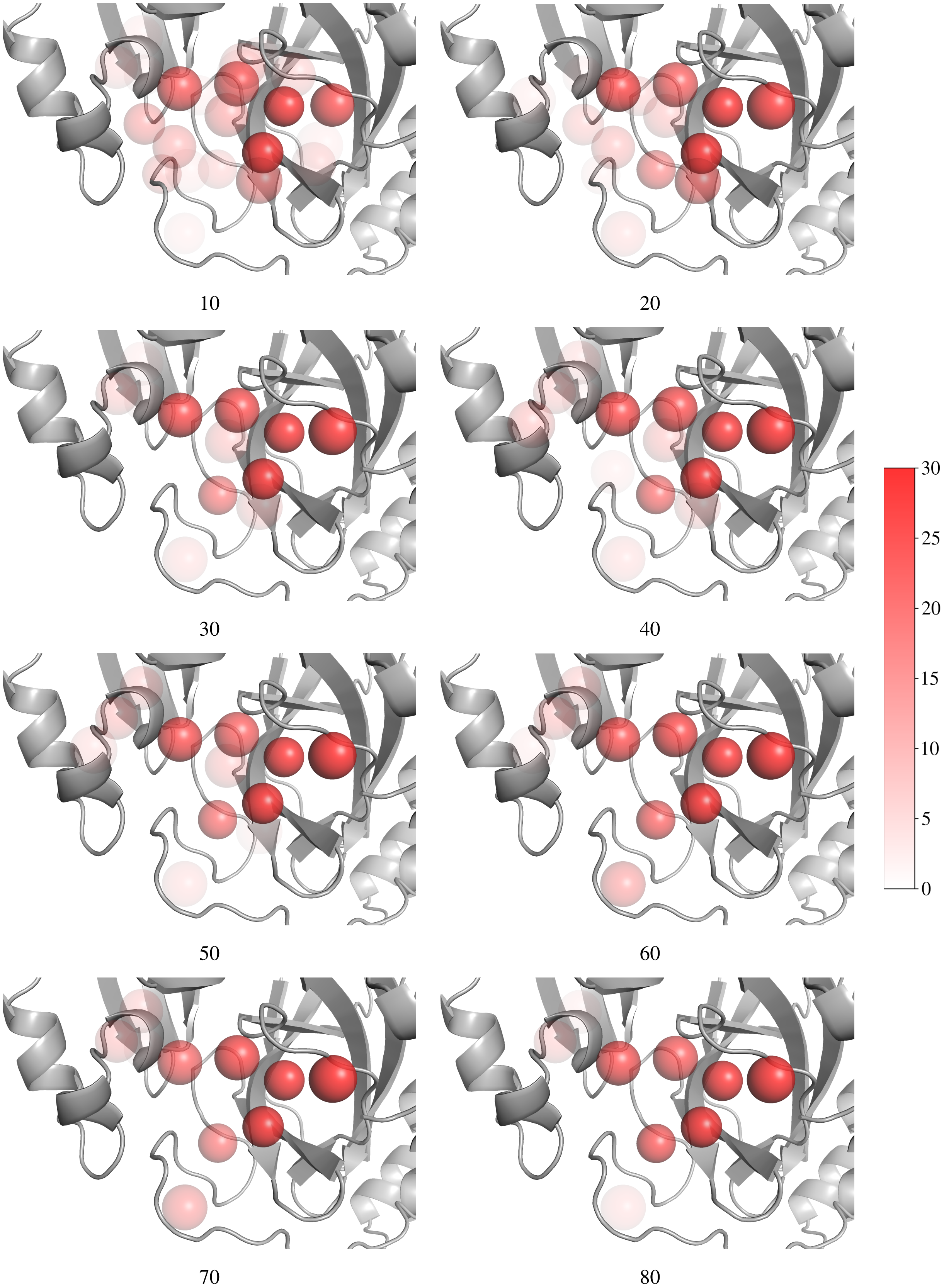

Fig. 4. Top 5 PointVS donor and acceptor hotspots (both shown in red) obtained in the pocket of Mpro by performing attribution on 40 randomly generated sets of bound fragments. The number of structures constituting each random set is shown below each image. Darker spheres correspond to hotspots which were included in the top 5 more frequently.

action sites in proteins," *J. Mol. Biol.*, vol. 289, no. 4, pp. 1093–1108, 1999.

[16] C. R. Groom, I. J. Bruno, M. P. Lightfoot, and S. C. Ward, "The Cambridge Structural Database," *Acta Crystallogr. Section B*, vol. 72, pp. 171–179, Apr 2016.

[17] T. E. Hadfield, F. Imrie, A. Merritt, K. Birchall, and C. M. Deane, "Incorporating target-specific pharmacophoric information into deep generative models for fragment elaboration," *Journal of Chemical Information and Modeling*, vol. 62, no. 10, pp. 2280–2292, 2022. PMID: 35499971.

[18] R. Skyner and F. von Delft, "Xchem, fragalysis." `https://fragalysis.diamond.ac.uk/`. Accessed: 2022-07-30.

[19] `https://fragalysis.diamond.ac.uk/viewer/react/download/tag/5740113a-7603-4af4-9523-7f902186d4a2`. Accessed: 2022-10-14.

[20] `https://fragalysis.diamond.ac.uk/viewer/react/download/tag/01f41754-b2bb-4817-acc3-a5ebe820316d`. Accessed: 2022-10-14.

[21] `https://fragalysis.diamond.ac.uk/viewer/react/download/tag/3df36d6b-3a5b-400d-97eb-af3b0e0df42d`. Accessed: 2022-10-14.

[22] C. W. Murray, D. R. Newell, and P. Angibaud, "A successful collaboration between academia, biotech and pharma led to discovery of erdafitinib, a selective fgfr inhibitor recently approved by the fda," *Med. Chem. Commun.*, vol. 10, pp. 1509–1511, 2019.

[23] H. Patani, T. D. Bunney, N. Thiyagarajan, R. A. Norman, D. Ogg, J. Breed, P. Ashford, A. Potterton, M. Edwards, S. V. Williams, G. S. Thomson, C. S. Pang, M. A. Knowles, A. L. Breeze, C. Orengo, C. Phillips, and M. Katan, "Landscape of activating cancer mutations in fgfr kinases and their differential responses to inhibitors in clinical use," *Oncotarget*, vol. 7, no. 17, pp. 24252–24268, 2016.
